# Evidence-based medicine training during residency: a randomized controlled trial of efficacy

**DOI:** 10.1186/1472-6920-10-59

**Published:** 2010-09-01

**Authors:** David A Feldstein, Matthew J Maenner, Rachaya Srisurichan, Mary A Roach, Bennett S Vogelman

**Affiliations:** 1 University of Wisconsin-Madison School of Medicine and Public Health, 310 N Midvale Blvd, Room 205, Madison, WI 53705 USA

## Abstract

**Background:**

Evidence-based medicine (EBM) has been widely integrated into residency curricula, although results of randomized controlled trials and long term outcomes of EBM educational interventions are lacking. We sought to determine if an EBM workshop improved internal medicine residents' EBM knowledge and skills and use of secondary evidence resources.

**Methods:**

This randomized controlled trial included 48 internal medicine residents at an academic medical center. Twenty-three residents were randomized to attend a 4-hour interactive workshop in their PGY-2 year. All residents completed a 25-item EBM knowledge and skills test and a self-reported survey of literature searching and resource usage in their PGY-1, PGY-2, and PGY-3 years.

**Results:**

There was no difference in mean EBM test scores between the workshop and control groups at PGY-2 or PGY-3. However, mean EBM test scores significantly increased over time for both groups in PGY-2 and PGY-3. Literature searches, and resource usage also increased significantly in both groups after the PGY-1 year.

**Conclusions:**

We were unable to detect a difference in EBM knowledge between residents who did and did not participate in our workshop. Significant improvement over time in EBM scores, however, suggests EBM skills were learned during residency. Future rigorous studies should determine the best methods for improving residents' EBM skills as well as their ability to apply evidence during clinical practice.

## Background

Competent clinical decision-making is a complex and critical process[1] and leaders in graduate medical education have long sought to hone residents' decision-making skills [2]. The introduction of evidence-based medicine (EBM) in 1992[3] and its subsequent inclusion by the Accreditation Council for Graduate Medical Education (ACGME) as a core component of practice-based learning and improvement[4] served as a catalyst for residency programs to incorporate evidence-based practice concepts into their curricula [5].

After two decades and a multitude of systematic reviews, non-randomized controlled studies, and pre- and post-intervention studies, the impact of formal EBM training on resident knowledge, skills, attitudes, and behavior remains unclear [6]. Challenges remain for translating EBM knowledge into clinical practice [7,8], and barriers to successful implementation of EBM skills have not yet been resolved [9]. Determination of the best methods for teaching clinical decision-making has been made difficult by the lack of well-validated evaluation tools and the absence of randomized controlled trials evaluating the impact of EBM educational interventions [10,11]. This paper reports results from a long-term, randomized controlled trial designed to test the hypothesis that participation in a brief interactive EBM workshop leads to increases in residents' EBM knowledge, literature searching, and self-reported use of evidence-based resources. Improvements in EBM competency are assessed across residency training in an effort to elucidate how best to prepare residents for effective clinical decision-making.

## Methods

### Participants and Setting

**Figure 1:**
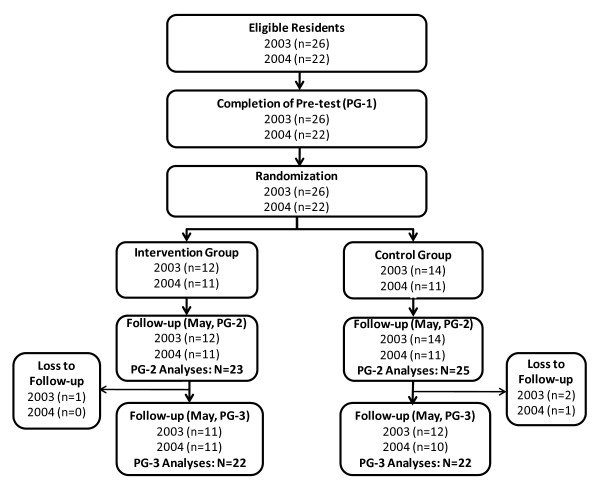
**Study design and flow of study participants**.

In May 2003 and May 2004, all categorical PGY-1 (postgraduate year 1) residents from two successive classes in the University of Wisconsin-Madison Internal Medicine Residency program completed an EBM knowledge and skills pre-test (Figure 1). Approximately half of the residents in each class were then randomized by computer-generated random numbers either to a treatment group (12 in 2003, 11 in 2004) where they participated in an EBM workshop during the fall of their PGY-2 year, or to a control group where they did not attend the workshop (14 in 2003, 11 in 2004). Six and 18 months later, in May of their PGY-2 and PGY-3 years, residents again completed EBM knowledge tests. This study protocol was approved by the University of Wisconsin Health Sciences Institutional Review Board. All residents received Institutional Review Board approved study information sheets prior to participation and provided implied consent by completing the EBM tests and surveys.

### Formal EBM Training

#### EBM Workshop

Residents in the treatment group participated in an interactive 4-hour EBM workshop, co-conducted by one faculty member (DF) and one librarian. The workshop, which took place in a computer lab, emphasized four steps in the EBM process: a) developing an answerable question, b) finding the best available evidence, c) evaluating the evidence, and d) applying the evidence to a patient care decision. The case-based teaching covered both therapeutic and diagnostic patient care decisions. Individual and group exercises focused on developing and refining clinical questions in the PICO (Patient, Intervention, Comparison, Outcome) format [12]; individually searching multiple evidence databases; evaluating individual articles for validity; calculating absolute risk reductions (ARR), numbers needed to treat (NNT), relative risk reductions (RRR) and likelihood ratios; and applying evidence to a patient care decision.

The only other formal EBM training that residents received during the study period was an EBM journal club. Each resident presented at journal club once during their PGY-3 year. They met with an advisor to develop a clinical question, and to search for and critically appraise the appropriate evidence. They presented the case and findings to other residents and internal medicine faculty during journal club.

### Outcome Measures

#### EBM Test

Residents were given 40 minutes to complete an EBM test of knowledge and skills that consisted of 25 multiple choice questions covering seven EBM focus areas: a) asking clinical questions, b) searching, c) EBM resources, d) critical appraisal of therapeutic and diagnostic evidence, e) calculating ARR, NNT, and RRR, f) interpreting diagnostic test results, and g) interpreting confidence intervals. The test items required application of EBM concepts. Each item was scored as either correct (1 point) or incorrect (0 points) for a maximum possible score of 25. The same test was used each year; all copies were collected immediately after completion and no feedback was provided to residents.

Our EBM test was developed by the first author (DF) in collaboration with a local EBM expert for a prior project with internal medicine residents [13]. It was revised based on item analysis to include five fewer questions and minor question rewording. Post-hoc construct validity was demonstrated by a one-way analysis of variance comparing the total EBM test scores of 10 first year medical students who had no previous exposure to EBM (*M *= 10.9, *SD *= 2.8) with the total EBM test scores of the 48 PGY-1 residents who participated in this study (*M *= 14.5, *SD *= 3.6) and 9 EBM experts (*M *= 22.9, *SD *= 2.1) who had served as teachers either in a national week-long EBM workshop or in a local EBM faculty development program. EBM experts earned significantly higher EBM test scores than PGY-1 residents (*p *< 0.001), who in turn, earned significantly higher scores than first year medical students (*p *= 0.004). Responsiveness of the test was also demonstrated with 16 practicing clinicians during a faculty development fellowship that included EBM training. Mean difference in fellows' pre-test to post-test EBM scores was 5.8 points (95% CI, 4.2, 7.4) [14].

#### Literature Searches and Resource Usage

In May of each year, at the time of EBM test administration, residents were asked to complete a brief questionnaire self-reporting the number of literature searches they performed during the past week and the number of times they used each of five evidence-based resources in the past month (irrespective of which service they were on): UpToDate, MEDLINE, ACP Journal Club, Cochrane Database of Systematic Reviews (CDSR), and Database of Abstracts of Reviews of Effects (DARE). These resources were freely available to residents at the time the study was initiated. The number of self-reported literature searches was categorized as 0, 1-2, 3-5, 6-10, or more than 10 times per week. The use of evidence-based resources was categorized as 0, 1-2, 3-5, 6-10, or more than 10 times per month.

#### Self-Efficacy and Workshop Evaluation

Residents who participated in the EBM workshop completed an anonymous 10-item self-assessment of their understanding and ability to practice EBM for therapeutic and diagnostic decision-making. They also responded to a single item evaluating the overall quality of the EBM workshop, rated on a 5-point Likert scale from strongly disagree (1) to strongly agree (5).

### Data analysis

Analysis of covariance (ANCOVA) was used to test the effect of treatment group on residents' EBM test scores in the PGY-2 and PGY-3 years, while controlling for baseline (PGY-1) EBM test scores as a covariate. T-tests were conducted to test for group differences in the change of mean test scores between time points. T-tests and Cohen's d were calculated to estimate the changes and effect sizes of EBM test scores for all residents over residency years. The median number of self-reported searches and resources used were computed for each group, and Mann-Whitney U tests were used to assess for differences between groups at each time point, as well as to test for differences between residency years. Descriptive analyses convey residents' self-assessments of knowledge gained following the EBM workshop. Data were analyzed using SPSS for Windows version 16 (SPSS Inc., Chicago, IL, USA).

## Results

### Baseline Characteristics and Participation

All 48 residents completed EBM pre-tests and questionnaires during their PGY-1 year and EBM post-tests and questionnaires during their PGY-2 year. Forty-four (92%) residents also completed EBM post-tests and questionnaires during their PGY-3 year. Baseline characteristics including PGY-1 EBM pre-test scores for all residents, number of searches, and resource usage are presented in Table 1. There were no statistically significant differences between the workshop and control groups at baseline.

**Table 1:** Baseline characteristics of PGY-1 residents

Characteristic	Workshop (*n *= 23)	Control (*n *= 25)
	*Mean *(*SD*)	
**PGY-1 EBM Pre-Test Scores**	13.7 (3.9)	15.3 (3.2)

	No. of residents (%)	
**Number of Searches (times per week)**		
0	7 (30%)	3 (12%)
1 - 2	8 (35%)	11 (46%)
3 - 5	6 (26%)	6 (25%)
6 - 10	2 (9%)	4 (17%)
**Resource Usage (in past month)**		
UpToDate	23 (100%)	25 (100%)
MEDLINE	18 (78%)	18 (72%)
ACP Journal Club	4 (17%)	9 (36%)
CDSR	3 (13%)	5 (20%)
DARE	0 (0%)	1 (4%)

There were no significant differences (p < 0.05) in baseline characteristics between workshop and control groups.

### Impact of Treatment Group on EBM Test Scores

There were no significant differences in mean test scores between treatment groups at either time point following the EBM workshop; the workshop group scored slightly--but not significantly--lower at both time points. Similarly, there were no significant differences in the amount each group improved over their baseline scores at each time point, as shown in Table 2. The difference in the change in mean test scores from PGY-1 to PGY-2 between the workshop group (mean score increase of 1.87) and control group (mean score increase of 0.88) was 0.99 (95% CI: -0.62, 2.60, t-test p-value = 0.221). For the PGY-1 to PGY-3 interval, the difference in the change in mean test scores was 0.41 (95% CI: -1.48, 2.29, t-test p-value = 0.664). After using ANCOVA models to adjust for baseline (PGY-1) scores, the differences between the workshop and control groups remained small at both time points. The adjusted mean score for the workshop group was 0.51 points higher (95% CI: -1.04, 2.05) than for the control group in the PGY-2 year (p = 0.510), and 0.07 points higher (95% CI: -1.80, 1.93) in the PGY-3 year (p = 0.944). Baseline test scores accounted for nearly all of the variance explained by the ANCOVA models (R^2 ^= 0.49 for PGY-2) and (R^2 ^= 0.42 for PGY-3), where low baseline scores were predictive of greater score increases at PGY-2 and PGY-3.

**Table 2:** EBM test scores by postgraduate level

	PGY-1			PGY-2				PGY-3			
	Mean	SD	n	Mean	SD	n	Change over PGY-1	Mean	SD	n	Change over PGY-1*
Workshop	13.65	3.88	23	15.52	3.95	23	1.87	17.09	3.89	22	3.00
Control	15.28	3.22	25	16.16	3.20	25	0.88	18.00	3.87	22	2.59
Combined Groups	14.50	3.61	48	15.85	3.55	48	1.35	17.55	3.86	44	2.80

*Calculated among residents participating at both time points; does not equal the difference of the means presented in the table due to participant attrition.

There was an overall increase in residents' mean EBM test scores over time. The mean score at PGY-2 was 1.35 points (95% CI: 0.55, 2.16) higher than at PGY-1 (p = 0.001), Cohen's *d *= 0.49. The mean score at PGY-3 was 2.79 points (95% CI: 1.86, 3.73) higher than at PGY-1 (p < 0.001), Cohen's *d *= 0.91.

### Literature searches and resource usage

Mann-Whitney U tests comparing residents in the treatment and control groups indicated similar numbers of literature searches during each of their three years of residency (*p *> 0.05) (see Table 3). In terms of EBM resource usage, PGY-2 residents in the treatment group reported more frequent usage of CDSR than residents in the control group (median monthly uses of 1-2 and 0, respectively, Mann-Whitney U *p *= 0.025), and slightly, but not significantly, higher usage of DARE (Mann-Whitney U *p *= 0.067). No differences were found in the frequencies of other resources used in the PGY-2 year; and treatment and control groups did not differ on any of the resources used during the PGY-3 year (*p *> 0.05).

**Table 3:** Literature searches and EBM resource usage per week, by treatment group and postgraduate level

	PGY-1 (n = 48)		PGY-2 (n = 48**)		PGY-3 (n = 44)	
	Group Median	p-value*	Group Median	p-value*	Group Median	p-value*
**Total Literature Searches**						
Workshop	1.5	0.25	4.0	0.50	4.0	0.48
Control	1.5		4.0		4.0	
						
**EBM Resource Usage**						
**Up To Date Searches**						
Workshop	10.0	0.46	> 10.0	0.35	> 10.0	0.37
Control	> 10.0		> 10.0		> 10.0	
						
**MEDLINE Searches**						
Workshop	1.5	0.65	8.0	0.77	6.0	0.68
Control	4.0		8.0		6.0	
						
**ACP Journal Club**						
Workshop	0.0	0.22	0.0	0.63	1.5	0.12
Control	0.0		0.0		1.5	
						
**CDSR**						
Workshop	0.0	0.66	1.5	**0.03**	1.5	0.74
Control	0.0		0.0		1.5	
						
**DARE**						
Workshop	0.0	0.34	0.0	0.07	0.0	0.71
Control	0.0		0.0		0.0	

*Mann-Whitney U p-value calculated

** Missing data for 1 resident on PGY-2 literature search item

PGY-3 residents reported a significantly greater number of literature searches than did PGY-1 residents: Median increase from 1-2 to 3-5 times per month (Mann-Whitney U *p *< 0.001). PGY-3 residents also exhibited significantly greater utilization of all EBM resources than they had as PGY-1 residents: Median MEDLINE access increased from 3-5 to 6-10 uses per month (*p *= 0.038); ACP Journal Club access increased from 0 to 1-2 uses per month (*p *< 0.001); DARE usage increased significantly (p <0.001), although the median usage was less than once per month at both PGY-1 and PGY-3 levels; and UpToDate usage increased significantly (*p *= 0.038), although residents generally reported using UpToDate >10 times per month at both time points.

### Self-efficacy and workshop evaluations

Residents' self-assessments of knowledge gained following the EBM workshop were consistently positive with average scores of 4.4 or above (on a 5-point Likert scale) in each of the following workshop content areas: Residents reported a better understanding of how to ask clinical questions about therapy (*M *= 4.5, *SD *= 0.5) and diagnosis (*M *= 4.4, *SD *= 0.7), how to search available databases for evidence about therapy (*M *= 4.9, *SD *= 0.4) and diagnoses (*M *= 4.8, *SD *= 0.4), and how to appraise the validity of therapeutic trials (*M *= 4.4, *SD *= 0.5) and interpret results of studies of diagnostic tests (*M *= 4.4, *SD *= 0.5). Residents consistently rated the workshop very highly (*M *= 4.5, *SD *= 0.5) and felt that participation in the workshop improved their ability to practice EBM (*M *= 4.5, *SD *= 0.5).

## Discussion

This randomized controlled trial assessed the impact of an EBM workshop on residents' EBM knowledge and skills within the context of residency training. Unlike previous short-term randomized [15] and non-randomized trials [12,16,17], the results failed to indicate an independent effect of our 4-hr interactive EBM workshop on residents' EBM test performance. Although residents rated the EBM workshop very positively and reported perceived gains in EBM knowledge, their perceptions were not associated with increases in EBM skills.

Several limitations could have prevented us from documenting an effect of our EBM workshop on residents' EBM test scores. First, although the difference in change in PGY-1 to PGY-3 test scores between the treatment and control groups was small (0.41), the confidence intervals around this change included a possible medium effect size, and the small sample size may have limited our ability to detect differences that might be considered clinically or educationally significant. Second, knowledge is a dynamic construct that changes over time [18-20]. Our assessments were conducted 6 and 18 months following the intervention, so short-term increases in EBM knowledge could have been missed, or masked by shared learning if workshop participants effectively translated their EBM knowledge to non-workshop participants through journal club, critical reading projects, and close interaction during clinical care. Third, although we did not assess residents' prior EBM training in medical school, we noted that our residents' baseline EBM test scores (58%) were actually much closer to the post-test scores reported by Smith et al.[21] (58-64%) and Fritsche et al.[16] (65%) than they were to the baseline scores reported in those studies (~40%). Although different tests were used in each study, it is possible that our residents' initially higher levels of EBM competency may have reduced their opportunities to demonstrate improvement.

Our results did, however, indicate significant improvement in residents' EBM knowledge, numbers of literature searches, and use of secondary evidence resources over the course of residency training. In addition, PGY-2 residents who participated in the workshop exhibited an increased use of CDSR and DARE compared with the control group. This is consistent with other randomized controlled trials demonstrating an increase in residents' use of evidence-based resources following EBM training [15] particularly when training focused on using secondary evidence resources instead of MEDLINE.

This study is one of only a few randomized controlled trials evaluating the impact of an EBM curriculum over the course of residency training. The fact that our results are inconsistent with some previous randomized controlled trials, for example, Kim et al. (2008), highlights the need for more rigorous, long-term trials of EBM education to provide guidance for implementing effective EBM programs. More intense doses of EBM education may be required to produce measurable improvement in residents' EBM skills [22].

Research also suggests that integration of EBM training into clinical practice may provide better results in improving EBM skills [10], although this has not been confirmed in randomized controlled trials. Furthermore, the true goal of EBM education is to improve resident behavior and patient outcomes. This will require a combination of educational interventions to teach basic skills, real time evidence access and incorporation of evidence into clinical care. Our data were collected from 2003-2006 and the change in the quality and availability of secondary resources since that time may require different methods of teaching and integration of EBM practices into clinical care.

Based on these results, our residency program has built a broader-based EBM curriculum that enhances faculty competencies, stimulates resident scholarship, and guides residents in their application of EBM at the point of care. We now provide separate interactive workshops for our PGY-1 and PGY-2 residents to allow time for reinforcement and consolidation of EBM skills. We are also integrating our EBM training into clinical care through piloting a new evidence-based "educational prescription" tool designed to walk residents through the steps of EBM while answering clinical questions at the point of care [23]. Validation of our evidence-based prescription tool as part of a multi-institutional trial will allow us to assess the generalizability of different types of EBM educational interventions and avoid issues of contamination that are common in single center studies [24].

## Conclusions

This randomized controlled trial was not able to detect a significant effect of a 4-hour EBM workshop on residents' EBM knowledge and skills, although residents did demonstrate an increase in EBM skills, literature searching, and resource usage over the course of residency training. Future multi-institutional randomized controlled studies are warranted to determine the best methods for improving residents' EBM skills as well as for enhancing their abilities to apply evidence during clinical practice.

## Competing interests

The authors declare that they have no competing interests.

## Authors' contributions

DAF conceived of the study, carried out the randomized controlled trial, participated in the analyses and drafted the manuscript. MJM participated in the statistical analyses and writing of the manuscript. RS participated in the statistical analyses and writing of the manuscript. MAR participated in the statistical analyses and writing of the manuscript. BV participated in the coordination of the study and in the writing of the manuscript. All authors read and approved the final manuscript.

## Pre-publication history

The pre-publication history for this paper can be accessed here:

http://www.biomedcentral.com/1472-6920/10/59/prepub
